# Construct and criterion validity of muscle ultrasonography for assessment of skeletal muscle in patients recovering from COVID-19

**DOI:** 10.3389/fphys.2023.1231538

**Published:** 2023-10-23

**Authors:** Kirby P. Mayer, Kate Kosmac, Yuan Wen, Selina M. Parry, Sanjay Dhar, Sarah Foster, Jonathan Starck, Ashley A. Montgomery-Yates, Esther E. Dupont-Versteegden, Anna G. Kalema

**Affiliations:** ^1^ Department of Physical Therapy, College of Health Sciences, University of Kentucky, Lexington, KY, United States; ^2^ Center for Muscle Biology, University of Kentucky, Lexington, KY, United States; ^3^ Department of Physiotherapy, School of Health Sciences, The University of Melbourne, Melbourne, VIC, Australia; ^4^ Division of Pulmonary, Critical Care, and Sleep Medicine, College of Medicine, University of Kentucky, Lexington, KY, United States; ^5^ Department of Biology, College of Arts and Sciences, University of Kentucky, Lexington, KY, United States

**Keywords:** muscle dysfunction, critical illness, post-intensive care syndrome, muscle ultrasound, skeletal muscle, muscle wasting, ICU-acquired weakness

## Abstract

**Background:** The purpose was to investigate the content, construct, and criterion validity of muscle ultrasound in a mixed cohort of participants recovering from mild and critical COVID-19.

**Methods:** A secondary analysis of a prospective cross-sectional study was conducted on data obtained from a battery of muscle and physical function assessments including a muscle biopsy and muscle ultrasonography (US). Rectus femoris (RF) muscle thickness (mT), quadricep complex (QC) mT, RF muscle cross-sectional area (CSA) using 2D freeform trace and estimated from Feret’s diameter, and RF echo intensity (EI) were assessed with US. Muscle fiber CSA, fiber type, protein content in muscle fibers, extracellular matrix content (ECM; wheat-germ agglutin), and percent area of collagen in ECM (picrosirius red) were examined from vastus lateralis muscle biopsies. Spearman rho correlations (r) were performed to assess validity of ultrasound parameters.

**Results:** Thirty-three individuals participated including 11 patients surviving critical COVID-19, 15 individuals recovering from mild-COVID, and 7 controls. There were several significant correlations between RF mT, QC mT, RF CSA, and RF EI with age, comorbid burden, body-mass index, and measures of muscle strength, muscle power, and physical function (range r = 0.35–0.83). RF Feret’s CSA correlated to CSA of type II muscle fibers (r = 0.41, *p* = 0.022) and the average size of all muscle fibers (r = 0.39, *p* = 0.031). RF EI was correlated with collagen in muscle ECM (r = 0.53, *p* = 0.003) and protein content in muscle tissue (r = −0.52, *p* = 0.012).

**Conclusion:** Muscle size and quality measured using US has moderate content and construct validity, and to lesser extent, fair to moderate criterion validity in a mixed cohort of individuals recovering from COVID. Muscle ultrasound quality (EI) appears to be sensitive at detecting muscle dysfunction as it is associated with strength, power, physical function, and collagen distribution in a mixed group of individuals recovering from COVID-19.

## Introduction

Skeletal muscle wasting and dysfunction are consequences of admission to the intensive care unit (ICU) for critical illness (Latronico and Guarneri, 2008; Puthucheary et al., 2010; Fazzini et al., 2023). Acute skeletal muscle deterioration is driven by the critical illness itself i.e., systematic inflammation, as well as the deleterious effects of treatment approaches i.e., immobilization and pharmaceutical interventions (Files et al., 2015; Parry and Puthucheary, 2015). Alterations in muscle size, architecture, and function that occur in the acute phase of illness contribute to short-and long-term impairments in physical function increasing the risk of morbidity and mortality in ICU survivors (Hermans et al., 2014; Puthucheary and Prescott, 2017; Mayer et al., 2020a; Medrinal et al., 2020). Therefore, assessment of skeletal muscle early and repeated across the spectrum of recovery will be meaningful for survivors of critical illness.

Muscle ultrasonography provides advantages over alternative methods of skeletal muscle assessment e.g., Computed Tomography and manual muscle testing (Mourtzakis et al., 2017), offering high clinical utility since it can be performed non-invasively in patients unable to follow commands. Moreover, healthcare professionals that have received bespoke muscle ultrasound training have strong-to-excellent reliability (Sarwal et al., 2015; Mayer et al., 2020b; González-Seguel et al., 2021). Ultrasonography is responsive to change over time with multiple studies demonstrating that patients suffer declines of 20%–30% of rectus femoris muscle size in the first 10 days of ICU admission for non-COVID (Puthucheary et al., 2013; Parry et al., 2015a; Mayer et al., 2020a) and COVID-19 (de Andrade-Junior et al., 2021) etiologies of critical illness. Muscle echo intensity (EI), a parameter derived from ultrasonography to examine muscle “quality,” was previously linked to muscle fiber necrosis in patients with critical illness of mixed etiologies (Puthucheary et al., 2015). Muscle characteristics derived from ultrasonography have also been correlated with MRI/CT measures of muscle mass (Paris et al., 2017; Lambell et al., 2021) as well as muscle strength and physical function at ICU discharge (Parry et al., 2015a; Palakshappa et al., 2018) and hospital discharge (Mayer et al., 2020a). The value of ultrasonography as a tool to examine skeletal muscle has led to an increase in utilization in critical illness research (Parry et al., 2015b; Connolly et al., 2015; Casey et al., 2022), but there are minimal data validating muscle ultrasound against morphological investigation of muscle tissue from biopsies. Moreover, muscle ultrasound has not been validated in the recovery phase of critical illness after hospital discharge, which is likely different than the acute phase of illness as skeletal muscles are no longer in state of catabolism (Puthucheary et al., 2013; Files et al., 2015; Puthucheary et al., 2015).

Research on long-term outcomes after critical illness demonstrates that patients have heterogeneous recovery patterns such that patients may better align with distinct phenotypes of muscle and physical recovery (Gandotra et al., 2019). Thus, ultrasonography has the potential to enhance the assessment of muscle health after hospital release to identify individuals who may benefit from targeted nutrition and exercise therapies (Heyland et al., 2016). We suggest that muscle ultrasound should be used by medical, rehabilitation, and nutrition clinicians in recovery of acute illness to enhance prognostication, track progress to interventions, and assist with identification on persistent muscular dysfunction (Mourtzakis et al., 2017; Wischmeyer et al., 2023). Therefore, the aim of this study was to determine: 1) criterion validity of ultrasound derived muscle quality compared to morphological assessment of skeletal muscle tissue and 2) examine the content and construct validity of muscle size and quality derived from ultrasound compared to muscle strength, power and physical function when assessed after acute illness. We hypothesized that higher EI (worse muscle quality) is strongly correlated to higher percentage of area of the muscle extracellular matrix (ECM) and percentage area of collagen in the ECM, which are considered reasonable gold standards (Emde et al., 2014). Secondarily, we hypothesized measures of muscle size derived from ultrasound are moderately correlated with muscle fiber size examined with morphological analyses, muscle strength, power, and physical function.

To enhance the ability to determine meaningful correlations of validity, we included a mixed cohort of individuals for two reasons: 1) enhanced range of observations providing a spectrum of health and muscular deficits (Janse et al., 2021); and 2) improved statistical power in mixed cohort when combining the small sample sizes common with studies including the muscle biopsies procedure (Comulada and Weiss, 2010). Thus, we investigated muscle ultrasound in a mixed cohort of individuals recovering from mild and critical COVID as well as community dwelling adults without illness. Secondarily, we explored the validity of ultrasound in the critical COVID survivors to provide preliminary evidence on ultrasound as a tool to detect muscle dysfunction in recovery phase to guide future investigations.

## Methods

### Study design

We performed a secondary analysis of a prospective, observational study from 23 March 2021 to 13 January 2022 The primary study was performed to examine biologic and morphologic characteristics of skeletal muscle in the recovery of critical illness compared to individuals with mild-COVID and community dwelling adults (controls) (*data are available upon reasonable request*). The primary analyses examined the clinical factors, morphological characteristics, and biological mechanisms driving or associating with long-term physical dysfunction in survivors of critical illness. ICU survivors participating in the primary study performed a battery of tests including examinations of muscle ultrasound and muscle biopsy. Subjects in the mild-COVID group and controls performed the same measures. For this secondary analysis, we combined participants into one group (*n* = 33) for a technical examination of the validity of ultrasonography-derived measures of skeletal muscle size and structure; we also explored the validity of ultrasonography in the critical illness cohort (*n* = 11) consistent with our overarching research focus.

### Ethical considerations and reporting guidelines

The study was approved by the medical internal review board at the University of Kentucky (MEDFL # 46072). Patients provided written informed consent before participation. This study was reported in accordance with the Strengthening the Reporting of Observational Studies in Epidemiology (STROBE) (von Elm et al., 2007) and Consensus-based Standards for the selection of Health status measurement instruments (COSMIN) guidelines (Mokkink et al., 2010).

### Patient populations

Adult participants (≥18 years of age) from three cohorts included in the primary study were examined in this secondary analysis:

1) Individuals recruited from the ICU Recovery Clinic following an ICU admission for COVID-19 (laboratory confirmed) who required mechanical ventilation (MV) via endotracheal tube, non-invasive MV, or high-flow nasal cannula (HFNC) (cCOVID); (Mayer et al., 2020c).
2) Individuals recovering from mild COVID (<120 days from positive testing) who did not require supplemental oxygen or hospitalization (mCOVID).
3) Community dwelling adults with no history of an admission to the ICU and reported not having COVID-19 in their health history (controls).

### Skeletal muscle and physical function assessments

All participants performed skeletal muscle and physical function testing at one time point at the University of Kentucky in the Center for Muscle Biology and the Center for Clinical and Translational Science, which occurred 6–12 months after hospital discharge for cCOVID and 60–120 days after positive test for mCOVID. The time-points were selected *a-priori* according to the original study.

Muscle ultrasound*:* The Sonosite IViz (FUJIFILM SonoSite Inc. Bothell, WA) portable ultrasound with 8.5-MHz linear transducer was used to assess the right quadriceps muscle size (cross-sectional area [CSA] and thickness [mT]), echo intensity (EI), and subcutaneous thickness. B-mode ultrasonography with auto-gain setting was performed with participants laying in supine with the lower-extremity in neutral rotation at the hip and ankle, the knee in passive extension, and the head-of-bed at 0°. Subjects unable to lay flat due to medical reason were permitted to have head-of-bed between 20 and 30°. Excess water-soluble ultrasound gel was applied to probe and anatomical landmark with sonographer using a minimal-to-no compression techniques to avoid depressing the dermis. Quadriceps muscle was imaged at 2/3 distance from Anterior Superior Iliac Spine to superior patella border with depth of 5.9 cm (Parry et al., 2015a; Puthucheary et al., 2015; Mourtzakis et al., 2017). Three images were acquired at the same landmark by resetting the probe to minimize potential distortion and artifacts. Device settings were kept constant between subjects with 1 experienced sonographer (KPM) acquiring all images. Images were stored and then transferred (hard-drive) to avoid image compression. Each ultrasound image was analyzed using ImageJ software (NIH, Bethesda, MD) (Rasband, 1997; Schneider et al., 2012) for quantification of following parameters: rectus femoris CSA measured in two approaches 2D freeform trace and 2d Ferret diameter CSA []), rectus femoris mT, quadriceps complex (rectus femoris and vastus intermedius [QC]) mT, subcutaneous and dermis thickness (SubQ-T), and rectus femoris EI using region of interest technique (Parry et al., 2015a; Sarwal et al., 2015). Freeform trace of CSA for rectus femoris muscle is based on the absolute area and common practice in ultrasonography (Parry et al., 2015a; Mayer et al., 2020b). However, the linear footprint of ultrasound probe may not capture the lateral horns of the muscle, potentially underestimating the CSA. Thus, 2D CSA is an estimate or representation of the muscle size (Mayer et al., 2020b). To address this issue, we suggest using Feret’s diameter which is an estimation of the cross-sectional area based on the longest distance between the two muscle planes (width and depth), commonly used in image analyses (Bianconi et al., 2023). Prior to image analysis, each image was coded by a research coordinator to blind the expert sonographer’s analysis. The average of each parameter from the three images were used in statistical analyses. The expert sonographer is a physical therapist-scientist (KPM) with >6 years of muscle ultrasonography experience (Mayer et al., 2020b). In addition, expert sonographer (SMP) provided oversight for approach, analyses, and dissemination of ultrasonography. The team has published and led multiple ultrasonography investigations (Parry et al., 2015a; Sarwal et al., 2015; Mourtzakis et al., 2017; Mayer et al., 2020a; Mayer et al., 2020b; Mayer et al., 2020d; González-Seguel et al., 2021).

⁃Muscular strength was assessed using 1) portable hand-held dynamometry for knee extensor strength (Lafayette Manual Muscle Test System Model-01165, Lafayette Company, Lafayette, IN) with assessment of muscle force production (kilograms) and the rate of force development (seconds). Assessment occurred in supine position with knee flexion 20–30° with a standard bolster (Bohannon, 1986; Stark et al., 2011) and 2) handgrip strength using JAMAR handgrip dynamometer (Parry et al., 2015c). All strength assessments occurred in triplicate bilaterally; with the average of the dominant side recorded for analyses.
⁃Lower-extremity muscle power was assessed with two measures: 1) muscle power was calculated based on the recorded velocity and distance of a unilateral (right) leg press (HUMAC-360, CSMi, Stoughton, MA). The average Watts of three repetitions of leg-press performed at two loads of resistance (2lbs and 21 lbs.) were used in the analyses; the testing procedures have been previously described (Mayer et al., 2020d); 2) Chair Rise Test to measure the time to perform 5 repeated sit-to-stand transfers without using the upper-extremities from standard height chair (17-inches) as a generalized assessment of lower-extremity muscle power; faster times indicating better performance (Alcazar et al., 2018; Mayer et al., 2020d).
⁃Physical function and functional exercise capacity were examined with performance of the Timed-up and Go-test (TUG) (ATS statement, 2002), the Short Performance Physical Battery (SPPB) (Parry et al., 2015d; Chan et al., 2016) and six-minute walk test (6MWT). TUG measures the time to stand from chair, walk 3-m and return to sitting with faster times indicating better functional status. (Needham et al., 2017) The SPPB is a 3-component assessment of function including 4-m habitual gait spend, Chair-Rise Test, and standing balance with max score of 12 (better functioning). (Parry et al., 2015d; Chan et al., 2016) The 6 MWT was performed as previously described following the American Thoracic Society standardized protocol examining the distance an individual can walk in six-minutes (ATS statement, 2002).
⁃Frailty: Clinical frailty scale (CFS) is a 9-point scale to quantify frailty scored by the physical-therapist after physical function testing (Brummel et al., 2020).
⁃Self-reported fatigue: Patients completed the FACIT-fatigue (0–52) with lower scores indicating worse perceived fatigue (Yellen et al., 1997).
⁃Muscle biopsy*:* Vastus lateralis muscle biopsies were obtained from participants using the Bergstrom needle technique previously described. (Tarnopolsky et al., 2011; Shanely et al., 2014) Muscle biopsies were performed on the same day about 1 hour after muscle and physical function tests were completed. Muscle tissues were prepared for frozen sectioning on a cryostat and immunohistochemistry (IHC) (Kosmac et al., 2018) and coded to blind scientists performing tissue analyses. Immunohistochemical analysis of muscle fiber CSA and type was carried out as previously described. (Murach et al., 2019) Briefly, 7 μm muscle cryosections were rehydrated with phosphate buffered saline and incubated overnight at 4°C with a primary antibody cocktail. Rabbit anti-laminin (L9393; Millipore-Sigma) was used to detect fiber borders, with the following isoform-specific myosin heavy chain (MyHC) antibodies: Type 1 (BA.D5; 1gG2b), Type 2a (SC.71; IgG1) and Type 2x (6H1; IgM) [Developmental Studies Hybridoma Bank (DSHB)]. Sections were then washed with PBS and incubated with fluorophore conjugated secondary antibodies: anti-rabbit AMCA (C1-1000, Vector Laboratories), anti-mouse IgG2b AlexaFluor 647 (A21242), anti-mouse IgG1 AlexaFluor 488 (A21121) and anti-mouse IgM AlexaFluor 555 (A21426) (ThermoFisher Scientific). Fiber size and type was quantified using MyoVision automated software (Wen et al., 2018). Wheat-germ agglutinin (WGA) was used to demarcate total muscle extracellular matrix (ECM) in cryosections following fixation with 4% paraformaldehyde. Sections were incubated with Texas Red-conjugated WGA (1 mg/mL at 1:50) (W21405, ThermoFisher) for 2 h. Picrosirius red (SR) was used to identify total collagen distribution in the muscle ECM in cryosections following fixation with 4% paraformaldehyde. Positive SR staining was determined in ImageJ by establishing a positive threshold and the percent of total area occupied by threshold was quantified. Bradford protein assays were completed to determine the concentration of protein (mg) per tissue (mg).

Demographic and clinical data ***:*** data extracted from the electronic medical record of the critical illness group including age, sex, body mass index (BMI), Charlson Comorbidity Index (CCI), mechanical ventilation utilization and duration, severity of illness in first 48 h of ICU admission per the Sequential Organ Failure Assessment (SOFA), ICU and hospital length of stay, and discharge disposition. For participants in mCOVID and control groups, demographic, symptoms, and COVID history were collected at the time of testing.

### Study outcomes

The primary outcome is the criterion validity of ultrasound derived muscle quality (EI) compared to morphologic assessment of skeletal muscle tissue examined with IHC analyses. EI derived from muscle ultrasound is supported as a measure of muscle quality, but evidence is limited and conflicting on the relationship between EI and underlying muscle composition (Stock and Thompson, 2021; Young et al., 2015; Pillen et al., 2009) increases in EI (worse quality) may be related to intramuscular adipocytes, fibrous tissues, and alterations in hydration or glycogen. In survivors of ICU, we hypothesize that EI is strongly correlated to higher percentage of area of the muscle ECM (WGA%) and percentage area of collagen in the ECM (SR%), which are considered reasonable gold standards for measuring ECM content (Emde et al., 2014). Secondary hypotheses included the determination of content and construct validity:

• *Content validity*: muscle ultrasound derived size (CSA, mT) will be moderately correlated with muscle fiber size examined with IHC analyses.
• *Construct validity:* muscle ultrasound derived size (CSA, mT) and quality (EI) will be moderately correlated with muscle strength, power, and physical function.
• *Construct validity:* muscle ultrasound derived size (CSA, mT) and quality (EI) will be moderately correlated with self-reported quality of life and fatigue, as well as clinician scores on the CFS.

### Sample size

*We performed a* post-hoc analysis of the final sample sizes of *n* = 33 (analyzed as a combined cohort of cCOVID, mCOVID, and controls) and *n* = 11 (critical illness only) which demonstrate that a moderate coefficient of 0.5 and alpha set at 0.05 yields the statistical power (1- Β err prob) equal to 0.85 and 0.34, respectively to detect a statistically significant correlation consistent with our hypotheses (G*Power 3.1, Fanz Faul, Universität Kiel, Germany) (Comulada and Weiss, 2010; Kang, 2021).

### Statistical analyses

Descriptive statistics were performed to examine the central tendencies and variance in the data as well as tested for normality using Shapiro-Wilk test. Mean and standard deviation or the median and interquartile range (IQR) are presented according to distribution. To examine the validity of muscle ultrasound, Spearman Rho analyses were performed with two approaches 1) entire cohort; and 2) the critical illness cohort. Correlation coefficients determined the bivariate relationship between muscle-derived parameters and assessment of muscle strength, power and physical function as well as examined with myofiber type and size, protein concentration, and ECM. Strength of the correlation coefficients were categorized as weak (0.10–0.24), fair (0.25–0.49), moderate (0.50–0.74), and excellent (0.75–1.0) with alpha level set at 0.05 (Overholser and Sowinski, 2008).

## Results

Thirty-three individuals participated in the study including 11 in cCOVID, 15 in mCOVID, and 7 controls. Muscle tissue was not obtained in one individual in critical illness group due to technical issues during the procedure and tissue from one cCOVID was unable to use for IHC analyses due to incorrect orientation of fibers during preparation, otherwise there were no missing data.

The median age of combined cohort (*n* = 33) was 41 (IQR 31–60). Participants were primarily male (64%) with median BMI of 31 (IQR 24–35). Demographic and clinical data stratified by the three cohorts are provided in Table 1. The critical illness cohort (*n* = 11) had a mean SOFA score at ICU admission was 10.5 ± 3.9 with median hospital LOS of 21 (IQR 11–29) days. Eight (73%) required invasive mechanical ventilation with median duration of 16 days (IQR 13–19), and the other three survivors required HFNC (Table 1). Patients in critical illness group participated in muscle testing a mean 267 ± 98 days after hospital discharge. Raw data for muscle strength, muscle power, and physical function are available from author upon reasonable request.

**TABLE 1:** Demographic, clinical, and muscle ultrasonography.

Parameter	Combined cohort (*n* = 33)	Controls (*n* = 7)	Mild-COVID (*n* = 15)	Critical illness (*n* = 11)
Demographics and Clinical Parameters				
Age, years, median [IQR]	41 [31–60]	60 [46–63]	31 [26–36]	56 [53–60]
Sex, female, n (%)	12 (36)	3 (42)	5 (33)	4 (36)
BMI, kg/m^2^, mean ± SD	27 ± 6.9	28 ± 3.6	25 ± 4.7	38 ± 5.9
CCI, mean ± SD	0 [0–2]	1 [0–3]	0 [0–0]	2 [1–3]
SOFA, mean ± SD				10.5 ± 3.9
APACHE-II, mean ± SD				28 ± 6.7
ICU LOS, median [IQR				17 [9–23]
Hospital LOS, mean ± SD				21 [11–29]
Muscle Ultrasonography Parameters				
Time to testing, days, mean ± SD^i^			55 ± 38	267 ± 98
Rectus femoris muscle thickness, cm, mean ± SD	1.58 ± 0.3	1.66 ± 0.3	1.71 ± 0.3	1.38 ± 0.3
Quadriceps complex muscle thickness, cm, mean ± SD	3.40 ± 0.7	3.39 ± 0.5	3.66 ± 0.6	3.04 ± 0.8
Rectus femoris CSA, cm^2^, mean ± SD	4.83 ± 1.1	5.26 ± 1.4	5.26 ± 1.0	4.16 ± 0.9
Rectus femoris Feret CSA, (d F), median [IQR]	3.60 ± 0.5	3.29 ± 0.8	3.78 ± 0.2	3.54 ± 0.3
Rectus femoris echo intensity, greyscale, mean ± SD	67 ± 29	70 ± 25	47 ± 18	90 ± 24
Subcutaneous thickness, cm, median [IQR]	1.0 ± 0.6	1.1 ± 0.7	0.78 ± 0.5	1.3 ± 0.6

BMI, body mass index; CCI, charlson comorbidity index; SOFA, sequential organ failure assessment; APACHE-II, Acute Physiology and Chronic Health Evaluation II; ICU, intensive care unit; LOS, length of stay; CSA, cross-sectional area.

i Time to participation in testing from hospital discharge for ICU survivors and from time of positive test (mild-COVID).

### Validity of muscle ultrasound in combined cohort

**FIGURE 1:**
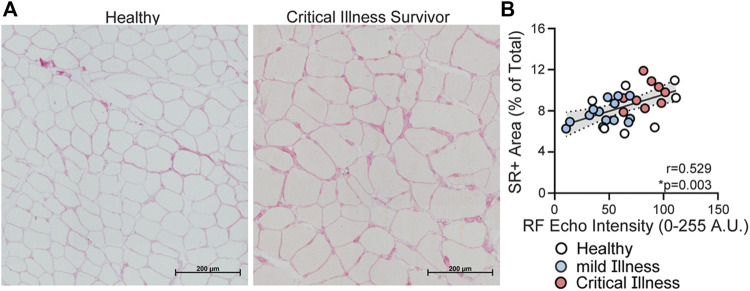
Relationship between rectus femoris echo intensity derived from ultrasonography and picrosirius red staining of muscle fibers. Panel **(A)** representative images of picrosirius red (SR+) area in muscle biopsy from a healthy community dwelling individual and an individual 9-months post critical COVID-19 (scale bar: 500 μm); Panel **(B)** Rectus femoris muscle EI is correlated with SR+ (r = 0.52, *p* = 0.003) in the combined cohort, with statistical significance attenuated in the critical illness group (r = 0.53, *p* = 0.194, red). Healthy community dwelling (*n* = 7) = white circles; mild COVID (*n* = 15) = blue circles; and critical COVID (*n* = 9) = red circles.

*Criterion Validity*: RF EI was positively correlated with total collagen percentage (SR+) in muscle ECM (r = 0.529, *p* = 0.003, Figure 1). Representative images showing SR + muscle area from a healthy individual and a patient surviving critical COVID are provided in Figures 1A, B. RF EI was not correlated with ECM content (WGA+).

**FIGURE 2:**
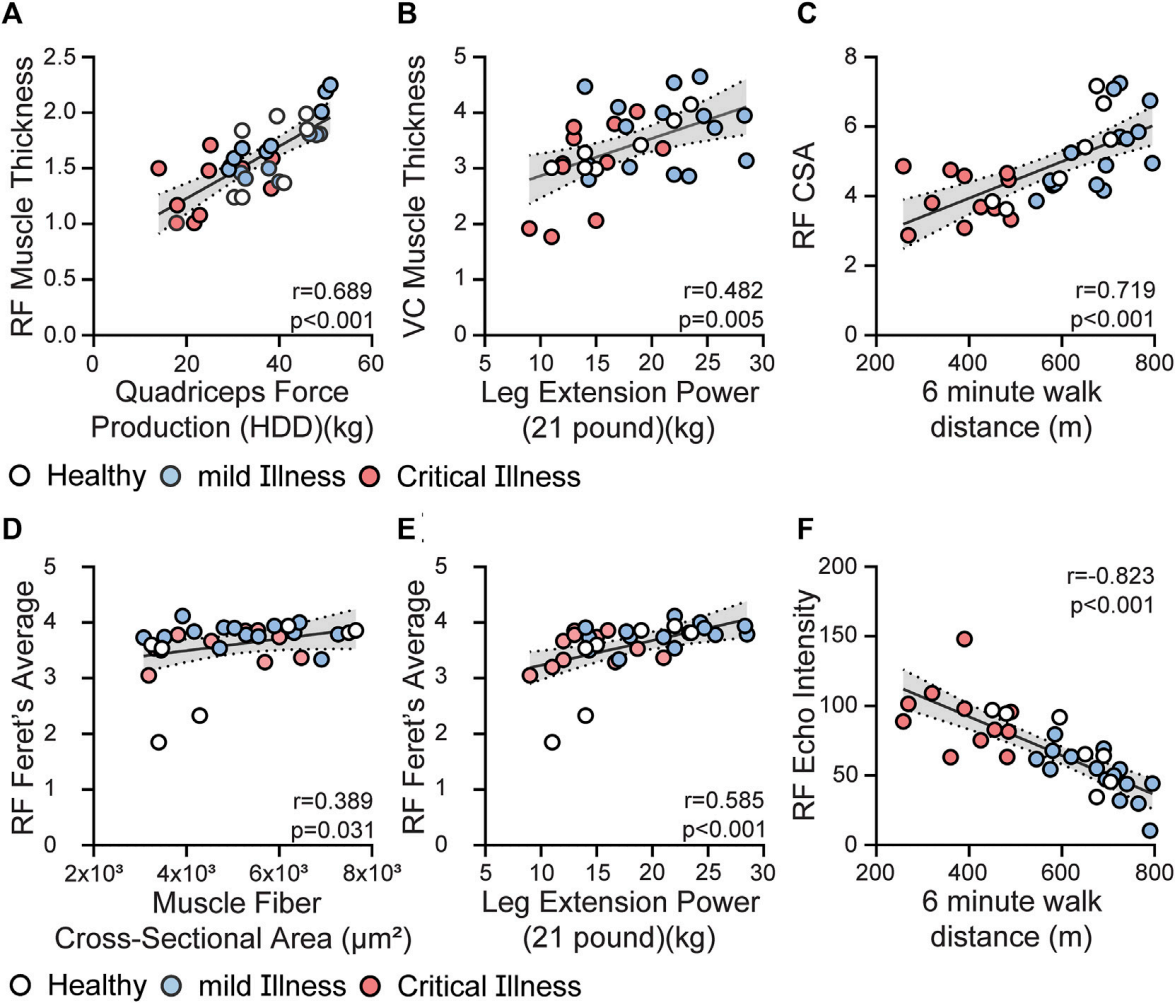
Relationship between US parameters and selected muscle strength and physical function testing. Panel **(A)** Rectus femoris muscle thickness is correlated with knee extensor strength, quadriceps force production, (r = 0.689, *p* < 0.001). Panel **(B)** Quadriceps complex thickness (rectus femoris and vastus intermedius thickness—VC) had moderate positive correlation with lower-extremity muscle power (r = 0.48, *p* = 0.005). Panel **(C)** RF 2D free-form CSA had positive moderate correlations with performance on 6 MWT (r = 0.72, *p* < 0.001). Panel **(D,E)** Rectus femoris Feret’s Diameter CSA is fair to moderately correlated with average CSA of muscle fibers from biopsy tissue (Panel D, r = 0.389, *p* = 0.031) and lower-extremity muscle power (Panel E, r = 0.585, *p* < 0.001). Panel **(F)** RF EI had negative strong correlation with performance on 6 MWT (r = −0.823, *p* < 0.001). Healthy community dwelling (*n* = 7) = white circles; mild COVID (*n* = 15) = blue circles; and critical COVID (*n* = 11 except Panel D *n* = 9) = red circles.

*Content Validity:* RF mT had a fair, positive correlation with protein in myofibers (r = 0.39, *p* = 0.08), and negative correlation with SR + area (r = −0.43, *p* = 0.016). QC mT had weak to fair correlations with SR + area (r = −0.34, *p* = 0.016), average CSA of all muscle fibers (r = 0.32, *p* = 0.083), and type II fiber CSA (r = 0.39, *p* = 0.032). RF Freeform CSA had moderate positive correlation with protein in myofibers (r = 0.45, *p* = 0.034) and fair negative correlation with SR + area (r = −0.34, *p* = 0.008). RF Feret CSA had weak to moderate positive correlations with the average CSA of all muscle fibers (r = 0.389, *p* = 0.031, Figure 2D) and type II fiber CSA (0.41, *p* = 0.022). RF EI had weak to fair negative correlations with protein in myofibers (r = −0.52, *p* = 0.08) and distribution of type 1 myofibers (r = −0.35, *p* = 0.008) as well as a negative fair correlation type 2ax myofibers (r = 0.34, *p* = 0.008).

*Construct Validity:* RF and QC mT were correlated to age, comorbid burden (CCI), BMI, as well as several measures of muscle strength, power and physical function. Data are presented in Table 2. Moderate positive correlations were observed between RF mT and QC mT with knee extensor force production (r = 0.6890, *p* < 0.001, Figure 2A; r = 0.74, *p* < 0.001, respectively), as well as muscle power (r = 0.482, p = 0.005, Figure 2B) Multiple statistically significant correlations were observed between both ultrasound measures of CSA with age, sex, BMI, and comorbid burden as well as measures of muscle strength and physical function; data are presented in Table 2. RF 2D free-form CSA had positive moderate correlations with performance on quadriceps strength (r = 0.71, *p* < 0.001) and 6 MWT (r = 0.72, *p* < 0.001, Figure 2C. RF Feret’s diameter had moderate (Figure 2E) positive correlation with muscle power (r = 0.585, *p* < 0.001). Multiple statistically significant correlations were observed between RF EI with age, sex, BMI, comorbid burden and muscle strength, power and physical function (Table 2), specifically RF EI had negative moderate to strong correlations with knee extensor strength (r = −0.69, *p* < 0.001), and, performance on 6 MWT (r = −0.823, *p* < 0.001, Figure 2F) as well as positive moderate to strong correlations with chair rise test performance (r = 0.68, *p* < 0.001) and CFS (r = 0.77, *p* < 0.001). SubQ-T was correlated with sex, BMI, and measures of muscle strength, muscle power and physical function presented in Table 2.

**TABLE 2:** Spearman correlation matrix in combined cohort ultrasound parameters with muscle and physical function outcomes.

Data are presented as r (*p*-value)^1^	Combined cohort					
	RF mT	QC mT	RF 2D CSA	RF F CSA	RF EI	SubQ-T
Age	−0.63 (<0.001)	−0.55 (0.001)	−0.57 (0.001)	−0.43 (0.013)	0.612 (<0.001)	0.28 (0.110)
*Sex*	−0.33 (0.063)	−0.40 (0.022)	−0.46 (0.008)	−0.52 (0.002)	0.47 (0.008)	0.78 (<0.001)
*BMI*	−0.40 (0.021)	−0.13 (0.457)	−0.50 (0.003)	−0.64 (<0.001)	0.63 (<0.001)	0.51 (0.003)
*Comorbid burden (CCI)*	−0.62 (<0.001)	−0.43 (0.012)	−0.60 (<0.001)	−0.44 (0.011)	0.67 (<0.001)	0.32 (0.077)
Muscle Strength and Power (*n* = 33)						
*Handgrip Strength*	0.57 (0.001)	0.62 (<0.001)	0.48 (0.005)	0.49 (0.007)	−0.54 (0.001)	−0.52 (0.002)
*Knee extensor HHD*	0.69 (<0.001)	0.76 (<0.001)	0.71 (<0.001)	0.61 (<0.001)	−0.69 (<0.001)	−0.54 (0.001)
*LE muscle power (2 lbs.)*	0.41 (0.018)	0.51 (0.003)	0.40 (0.022)	0.46 (0.007)	−0.50 (0.003)	−0.47 (0.006)
*LE muscle power (21 lbs.)*	0.43 (0.019)	0.48 (0.005)	0.41 (0.153)	0.59 (<0.001)	−0.52 (0.002)	−0.48 (0.005)
*Chair Rise Test (sec)*	−0.49 (0.012)	−0.44 (0.011)	−0.56 (0.001)	−0.56 (0.001)	0.68 (<0.001)	0.47 (0.006)
Physical Function and Fatigue (*n* = 33)						
*4* *m gait speed*	−0.12 (0.500)	−0.14 (0.447)	−0.25 (0.162)	−0.47 (0.006)	0.25 (0.014)	0.24 (0.172)
*SPPB*	0.41 (0.017)	0.35 (0.049)	0.49 (0.004)	0.49 (0.004)	−0.60 (<0.001)	−0.33 (0.061)
*6 MWT*	0.63 (<0.001)	0.47 (0.005)	0.72 (<0.001)	0.66 (0.210)	−0.82 (<0.001)	−0.63 (<0.001)
*Percent 6MWT*	−0.35 (0.043)	−0.31 (0.085)	−0.15 (0.420)	0.03 (0.853)	0.25 (0.162)	0.01 (0.979)
*TUG (sec)*	−0.30 (0.090)	−0.22 (0.207)	−0.35 (0.045)	−0.38 (0.032)	0.51 (0.002)	0.36 (0.043)
*CFS*	−0.57 (<0.001)	−0.44 (0.010)	−0.66 (<0.001)	−0.59 (<0.001)	0.77 (<0.001)	0.50 (0.003)
*FACIT-Fatigue (0–52)*	0.36 (0.039)	0.20 (0.273)	0.44 (0.011)	0.37 (0.035)	−0.43 (0.012)	−0.34 (0.051)
Immunohistochemical and histochemical analyses of muscle tissue (*n* = 31)						
*Protein conc*	0.38 (0.029)	0.13 (0.580)	0.45 (0.034)	0.28 (0.211)	−0.52 (0.012)	−0.37 (0.087)
*Fiber Type Frequency*						
*Type I*	0.38 (0.035)	0.37 (0.040)	0.35 (0.057)	0.23 (0.209)	−0.35 (0.055)	−0.07 (0.684)
*Type IIa*	0.10 (0.589)	0.17 (0.38)	0.12 (0.528)	0.02 (0.923)	0.10 (0.585)	−0.03 (0.880)
*Type IIa/x*	−0.50 (0.004)	−0.43 (0.015)	−0.52 (0.003)	−0.22 (0.240)	0.34 (0.058)	0.19 (0.316)
*Fiber Size* (*CSA, µm* ^*2*^)						
*Type I*	0.09 (0.627)	0.16 (0.384)	0.05 (0.783)	0.21 (0.278)	−0.18 (0.343)	−0.40 (0.027)
*Type IIa*	0.21 (0.248)	0.36 (0.048)	0.19 (0.314)	0.35 (0.052)	−0.19 (0.299)	−0.37 (0.041)
*Type IIa/x*	0.20 (0.292)	0.31 (0.093)	0.11 (0.567)	0.28 (0.122)	−0.22 (0.244)	−0.38 (0.024)
*Mean of all fibers*	0.18 (0.347)	0.32 (0.083)	0.16 (0.470)	0.39 (0.031)	−0.19 (0.305)	−0.45 (0.011)
*Extracellular Matrix (WGA+)*	−0.35 (0.057)	−0.39 (0.032)	−0.34 (0.060)	−0.24 (0.162)	0.19 (0.295)	0.17 (0.360)
*Collagens (SR+)*	−0.43 (0.016)	−0.34 (0.066)	−0.48 (0.008)	−0.56 (0.001)	0.53 (0.003)	0.36 (0.051)

RF, rectus femoris; QC, quadriceps complex; EI, echo intensity; mT = muscle thickness; subQ-T, subcutaneous thickness; RF F CSA, rectus femoris Feret’s diameter cross-sectional area; RF, 2D CSA, rectus femoris 2-dimenisonal freeform CSA; HHD, handheld dynamometry; LE, lower extremity; 6 MWT, 6 minute walk test (distance); TUG, timed up and go test; CFS, clinical frailty scale; WGA% = percent area of Wheat germ agglutin; conc, = concentration.

i Data are presented with spearman rho correlation (r) listed first followed by the *p*-value listed second inside the parentheses. Green shading is provided to highlight statistical significant correlations; dark green shading depicts significance level of <0.001; and light green shading at significance level of 0.05 to 0.001.

### Exploratory validity of muscle ultrasound in cCOVID

RF EI was positively correlated, although not statistically significant, with total collagen percentage (SR+) in muscle ECM of cCOVID patients (r = 0.53, *p* = 0.194). Moderate positive correlations were observed between RF mT and QC mT with knee extensor strength when examined only in c-COVID group (r = 0.59, *p* = 0.061; r = 0.77, *p* = 0.007, respectively). RF EI had a negative moderate correlation with knee extensor strength (r = −0.69, *p* = 0.052) and moderate positive correlation time to complate chair rise test (r = 0.68, *p* 0.044) in cCOVID group (higher EI, worse quality, correlated with longer time to complete the test).

## Discussion

The assessment of muscle characteristics via ultrasonography in a mixed cohort of individuals recovering from COVID has fair to moderate strength of content, construct, and criterion validity of muscle health and function. Of note, we present data to demonstrate that ultrasonography analyses of muscle size (mT and CSA) and quality (EI) were correlated to IHC of muscle tissue, muscle strength, and physical function. We also demonstrate a few significant correlations in the critical COVID group even despite being underpowered due to small sample sizes. The findings demonstrate that larger mT and CSA as well as lower EI (“better quality”) is related to better performance on muscle and physical function testing. supporting the content and construct validity of muscle ultrasonography. Our data suggest that muscle EI measured by ultrasound has moderate criterion validity when examined with measurements of muscle morphology at the tissue level. The findings demonstrate that hyperechoic areas of the imaged quadriceps muscle may in part be explained by alterations in collagen in the muscle ECM. Our study provides evidence that muscle ultrasonography provides a valid non-invasive approach for assessment of muscle size, quality, and function.

The purpose of the study was not to compare outcomes among groups, but rather examine psychometric properties of muscle ultrasonography. We included controls and individuals recovering from mild-COVID to enhance the spectrum of muscle size and quality as well as provide enough statistical power to examine correlative outcomes. Important for clinical interpretation, the timing of testing for the COVID groups were different and selected based on early reports from pandemic about the potential for long-term deficits. Testing in the mild group (∼2-3-months post initial infection) was selected to capture prolonged muscle deficits with 12-weeks being utilized for diagnosis of post-acute sequelae of COVID (Dražić et al., 2016; Nielsen et al., 2022).Testing in the critical group was selected based on previous non-COVID literature in ICU survivors suggesting that muscle deficits persist for months after discharge (Sponbeck et al., 2021). It should be noted that there were expected differences in the demographics, specifically age and BMI, but when analyzed as a combined cohort the variance may enhance the generalization to other patient populations. Moreover, the diversity of included patients may enhance our conclusions as we demonstrate statistically significant correlations despite the heterogeneity. We recognize this may also be viewed as a limitation as participants enrolled could be at risk of selection and representation biases (i.e., participants with transportation issues or patients on high dose anti-coagulant declined or could not participate). Additionally, participants in the controls may not be “healthy” as multiple individuals had pre-existing comorbidities. Clinicians and researchers should consider the strengths and weaknesses of the study, but we suggest the ultrasonography parameters represent and mirror clinical practice as heterogeneity is expected in human trials.

The findings demonstrate that muscle size determined from ultrasound images is fairly to moderately related to muscle fiber CSA from IHC analyses. These assessments support the content and criterion validity of ultrasound compared to muscle biopsy which is sometimes viewed as gold-standard for muscle size assessment. In addition, our data suggest the CSA estimated from Feret’s diameter and the 2D freeform CSA approach have similar value. We initially hypothesized that Feret’s diameter may improve assessment due to the irregular shape of the 2D traces (Sponbeck et al., 2021) with the potential not to capture the lateral horns of the quadriceps muscle in the image due to the linear probe footprint. CSA estimated from Feret’s diameter may account or address for such variations in imaging, however, we demonstrate that both approaches to assessing CSA with US hold value.

Previous literature demonstrates that ultrasound-derived measures of CSA are correlated with MRI in healthy individuals (Sponbeck et al., 2021; Naruse et al., 2022). Ultrasound images and muscle biopsies performed in thirty patients in the ICU previously demonstrated that rectus femoris echo intensity was related to development of muscle fiber necrosis in vastus lateralis muscles (Puthucheary et al., 2015). The authors acknowledge that “muscle necrosis appears anatomically patchy” which may address subtle clinical variations such as ultrasonographic waves and window not capturing histochemical changes observed in muscle fibers. We did not perform pathological assessment to quantify necrosis as patients were 9-months post discharge and no longer in the active state of wasting. We do, however demonstrate that higher EI (“worse quality”) is related to an increase in collagen in muscle ECM. SR is an accepted standard to visualize and quantify collagen distribution in muscle fibers (Vogel et al., 2015). Contrary to our initial hypothesis, WGA was not statistically significantly correlated to RF EI. WGA binds to glycoproteins of cell membranes which stains all contents of the ECM of the muscle. Thus, we suggest that change in EI is perhaps related to collagen in the ECM. Finally, RF EI had multiple significant relationships with muscle strength, power, and physical function as well as age, BMI and comorbid index, reiterating that lower EI (muscles appearing hypoechoic) is indicative of better quality and a valid indicator of physical function.

The study is not without limitations. Primarily, the sample sizes limit the statistical power limiting our ability to perform statistical analyses in subgroups. We did perform correlative tests in the cCOVID group as exploratory. The sample sizes, however, are consistent with previously published muscle biopsies studies in ICU survivors (Angel et al., 2007; Dos Santos et al., 2016; Walsh et al., 2016). The sample sizes may also be viewed as a strength regarding the inclusion of muscle biopsy (*n* = 31). Importantly, we reported the biopsy failure (one patient, 3%) and technical difficulty with IHC (one patient, 3%), both occurring in the critical COVID survivors, which has previously been highlighted as a potential limitation in research studies of frail individuals (Wilson et al., 2018). Failure to obtain tissue as well as aforementioned selection and representation biases may reduce the ability to generalize our findings. The SOFA and APACHE-II scores at time of ICU admission demonstrate a high severity of initial illness in the critical illness cohort, and we suggest our findings strongly represent survivors of critical COVID-19 occurring roughly 9-months after discharge. It may be argued that the selection of muscle for ultrasound (RF and vastus intermedius) and biopsy (vastus lateralis) are incongruent and differences in muscle fiber type may reduce the strength of the argument, however, the selection of muscles for biopsy and US mirror current standards in practice. Moreover, the approach to reiterative correlative testing may be viewed as a limitation leading to potential Type I errors. Finally, the selection of muscle and physical function assessments were, again, based on clinical practice, but may impart ceiling effects, especially in younger, community dwelling adults. The ceiling effects with these measurements have a high potential to reduce the strength of the correlations, and should be give consideration when interpreting our findings.

## Conclusion

Muscle size and quality (EI) derived from ultrasound are moderately correlated muscle strength, power, physical function, and measures of muscle extra-cellular matrix. Therefore, ultrasound can be used as a non-invasive method to assess muscle health in patients recovering from mild and critical illnesses.

## Data Availability

The raw data supporting the conclusion of this article will be made available by the authors upon reasonable request, without undue reservation.
